# A Model for Non-Arrhenius Ionic Conductivity

**DOI:** 10.3390/nano9060911

**Published:** 2019-06-24

**Authors:** Masaru Aniya, Masahiro Ikeda

**Affiliations:** 1 Department of Physics, Faculty of Advanced Science and Technology, Kumamoto University, Kumamoto 860-8555, Japan; 2 Department of General Education, National Institute of Technology, Oita College, Oita 870-0152, Japan; mikeda@hotmail.co.jp

**Keywords:** non-Arrhenius, ionic conductivity, size effect, Bond Strength–Coordination Number Fluctuation model, BSCNF model, solid electrolytes, ionic liquids

## Abstract

Non-Arrhenius ionic conductivity is observed in various solid electrolytes. The behavior is intriguing, because it limits the magnitude of ionic conductivity at high temperatures. Understanding the nature of this behavior is of fundamental interest and deserves attention. In the present study, the temperature dependence of the ionic conductivity in solids and liquids is analyzed using the Bond Strength–Coordination Number Fluctuation (BSCNF) model developed by ourselves. It is shown that our model describes well the temperature dependence of ionic conductivity that varies from Arrhenius to non-Arrhenius-type behavior. According to our model, the non-Arrhenius behavior is controlled by the degree of binding energy fluctuation between the mobile species and the surroundings. A brief discussion on a possible size effect in non-Arrhenius behavior is also given. Within the available data, the BSCNF model suggests that the size effect in the degree of the non-Arrhenius mass transport behavior in a poly (methyl ethyl ether)/polystyrene (PVME/PS) blend is different from that in a-polystyrene and polyamide copolymer PA66/6I.

## 1. Introduction

Ionic conduction in solids is one of the physical phenomena widely used in modern technology. It is a key phenomenon operating in batteries, a device that our everyday life depends on. However, from the fundamental science point of view, we still have not gained a satisfactory understanding of the physical phenomena occurring in battery materials. One of these phenomena is non-Arrhenius ionic conductivity, the topic of the present paper.

Usually, the temperature dependence of the ionic conductivity in solids exhibits an Arrhenius-type behavior. Specifically, when the logarithm of the conductivity is plotted versus the inverse of temperature, the data follows a straight line. Owing to this behavior, the expression based on the Arrhenius equation together with the Nernst–Einstein relation and the transition state theory (or the kinetic theory) has often been applied to describe the ionic conduction in solids, where the activation entropy and enthalpy terms are assumed to be temperature independent. However, some years ago, it was discovered that the ionic conductivity in optimized ionic conducting glasses exhibits a non-Arrhenius-type behavior [1]. The conductivity of these glasses follows the Arrhenius law at low temperature, while it deviates gradually from the straight line as the temperature increases, and at high temperature the conductivity seems to saturate, thus exhibiting a non-Arrhenius ionic conductivity behavior as a whole. This experimental finding has attracted much interest from both fundamental and application points of view. From a fundamental aspect point of view, the continuous transformation of the conductivity from an Arrhenius to a Vogel–Fulcher–Tammann (VFT) pattern is quite interesting, because it reflects the presence of complex physical processes. It has been also suggested that the non-Arrhenius behavior of ionic conductivity is a characteristic feature of fast ionic conducting glasses having optimized chemical composition [1]. This suggestion has strong implications for application because it limits the value of the ionic conductivity at high temperatures, which could be a serious obstacle in the use of solid electrolytes in devices. Currently, non-Arrhenius ionic conductivity is observed in various types of ionic conductors [2,3,4,5,6,7,8,9,10,11,12,13,14,15,16,17,18,19,20,21]. Perhaps the deviation from the Arrhenius behavior could be a universal behavior of good ionic conductors irrespective of the material being crystalline or amorphous.

Motivated by the pioneering work carried out in [1], a large number of theoretical and experimental studies have been done to understand the peculiar behavior of non-Arrhenius ionic conductivity [22,23,24,25,26,27]. We have also proposed theoretical models to describe and understand the behavior [25,26]. In the present paper, after giving a short review on our previous work regarding the description of non-Arrhenius-type ionic conduction, we present new results related to the subject. A description of the non-Arrhenius phenomenology over a wide temperature range is the first step to a deeper understanding of the property. In that sense, it is beneficial to describe the ionic conductivity in liquids, in addition to the case of solids. In this paper, we show that the ionic conductivity in both solids and liquids can be described based on similar mathematical expressions that are derived from a common physical model [28,29]. Related to the subject, we want to point out that the model has been used in the study of mechanical properties around the liquid–glass transformation temperature [30].

In the last few decades, many studies have been done regarding the size effects in the properties of materials [31,32,33]. Some of these properties have strongly impacted the materials’ applications. Other properties have motivated the search for new physical concepts. In relation with the present study, the size effect in ionic conductivity has also been studied [32]. However, as far as we are aware, the size effect in non-Arrhenius ionic conductivity remains unexplored. In the last part of this paper, a discussion on possible size effect in non-Arrhenius behavior is given based on notions gained from ongoing and other studies.

## 2. Model of Non-Arrhenius Ionic Conductivity

### 2.1. Ionic Conductivity in Solids

As described briefly in the introduction, the non-Arrhenius temperature dependence of ionic conductivity is considered to be a ubiquitous feature of fast ionic conductors [1] whose mechanisms have not been fully understood. In an early study done in our group, an analytical model for non-Arrhenius behavior was constructed by using the Zwanzig model of diffusion [34]. There, it was shown that the deviation from the Arrhenius behavior starts to be notable when the lifetime of the oscillating particle in a potential well becomes comparable with the inverse of oscillation frequency [25]. Such a model was applied to different materials. The analysis indicated that the non-Arrhenius behavior results from the interplay between carrier generation and relaxation processes [25]. It is worth noting that such a model indicates that low oscillating frequency is favorable for ionic conduction when the conductivity follows an Arrhenius-type behavior.

More recently, we developed another model for non-Arrhenius ionic conductivity [26] inspired by the Bond Strength–Coordination Number Fluctuation (BSCNF) model of the viscosity proposed by ourselves [28,29]. In the following sections, we focus on this model. According to the model, the diffusing ion is assumed to oscillate in a potential well formed by the surrounding ions. Over time, some of these ions can escape from the potential well with a certain probability. When the mobile ion overcomes the potential barrier by hopping to an adjacent site, the bonds connecting the oscillating ions to the surrounding components are broken or twisted. The series of such processes are mediated by the bond-breaking which is triggered by thermal fluctuations.

The mean residence time of the mobile ions in the potential well based on the above picture can be expressed as follows [26]:

(1) $$\tau =\frac{1}{\nu }\overset{\infty }{\underset{-\infty }{\int }}\overset{\infty }{\underset{-\infty }{\int }}d\tilde{E}d\tilde{Z}f\left(\tilde{E}\right)g\left(\tilde{Z}\right)\exp\left(\frac{\tilde{E}\tilde{Z}}{RT}\right),$$

where $\tilde{E}$ is the bond energy between the mobile and the surrounding ions, $\tilde{Z}$ is the coordination number of the mobile ion, $f\left(\tilde{E}\right)$ and $g\left(\tilde{Z}\right)$ are the distribution functions of $\tilde{E}$ and $\tilde{Z}$, ν is the oscillation frequency of the ion trapped in the potential well, *T* is the temperature, and *R* is the gas constant. By adopting Gaussian distributions for $f\left(\tilde{E}\right)$ and $g\left(\tilde{Z}\right)$, the above expression reduces to the following:

(2) $$\tau =\frac{\nu^{-1}T}{\sqrt{T^{2}-{\left(\Delta \tilde{E}\Delta \tilde{Z}/R\right)}^{2}}}\exp\left(\frac{{\tilde{E}}_{0}{\tilde{Z}}_{0}/R}{T-\Delta \tilde{E}\Delta \tilde{Z}/R}\right),$$

where ${\tilde{E}}_{0}$ and ${\tilde{Z}}_{0}$ are the mean values of $\tilde{E}$ and $\tilde{Z}$, and $\Delta \tilde{E}$ and $\Delta \tilde{Z}$ are their fluctuations. Equation (2) was derived under the assumption that $\Delta \tilde{E}/{\tilde{E}}_{0}=\Delta \tilde{Z}/{\tilde{Z}}_{0}$. When this condition is satisfied, Equation (1) reduces to the VFT-like expression given in Equation (2).

The expression for the conductivity can be obtained by replacing τ given above into the expression of the diffusion coefficient,

(3) $$D=\frac{gl^{2}}{\tau },$$

and the Nernst–Einstein relation,

(4) $$\sigma =\frac{{\left(Ze\right)}^{2}nD}{fk_{B}T}.$$

Here, g is a geometrical factor, l is the jump distance, Ze is the charge of the ion, f is the correlation factor, and n is the concentration of mobile ions. The derived expression of the ionic conductivity is as follows:

(5) $$\sigma T=\frac{A_{\sigma T}\sqrt{T^{2}-{\left(\Delta \tilde{E}\Delta \tilde{Z}/R\right)}^{2}}}{T}\exp\left(-\frac{{\tilde{E}}_{0}{\tilde{Z}}_{0}/R}{T-\Delta \tilde{E}\Delta \tilde{Z}/R}\right),$$

where

(6) $$A_{\sigma T}=\frac{g\nu {\left(Ze\right)}^{2}l^{2}n}{fk_{B}}.$$

In the literature, we find different expressions for ionic conductivity. Regarding the pre-exponential factor, the expression of σ given by Equation (5) is proportional to 1/T. It comes from the Nernst–Einstein relation which assumes a simple temperature-independent jump process. In some works [5,14], the pre-exponential factor does not include the temperature. Formally, this arises from the application of transition state theory under the assumption that the vibrational degree of freedom is excited [35]. We also find expressions where the pre-exponential factor has a $T^{-1/2}$ dependence [36]. This arises from an application of the kinetic theory of gases [35]. From a practical point of view, the explicit form of the pre-exponential factor’s temperature dependence is frequently omitted, because this term is usually much smaller than the exponential part [37]. Of course, from a fundamental point of view, the explicit form has meaning.

Usually, the expression of ionic conductivity is written in terms of temperature-dependent carrier concentration and mobility [35,38]. In those cases, the carrier concentration is written in terms of formation enthalpy $\Delta H_{f}$ and formation entropy $\Delta S_{f}$, and the mobility is written in terms of migration enthalpy $\Delta H_{m}$ and migration entropy $\Delta S_{m}$ [35,38]. In order to interpret the data by using the usual expression, we need to know these quantities. In our model, the diffusivity D corresponds to the mobility, and the temperature dependence of carrier concentration is not taken into account explicitly. Instead, the effect of the formation and migration enthalpies and entropies are incorporated in the values of $\tilde{E}$ and $\tilde{Z}$ by considering their distributions. Since $\tilde{E}$ and $\tilde{Z}$ have clear physical meaning and are easy to visualize, we believe that our approach provides an alternative way of interpreting the mass transport data in addition to the traditional approach.

**Figure 1:**
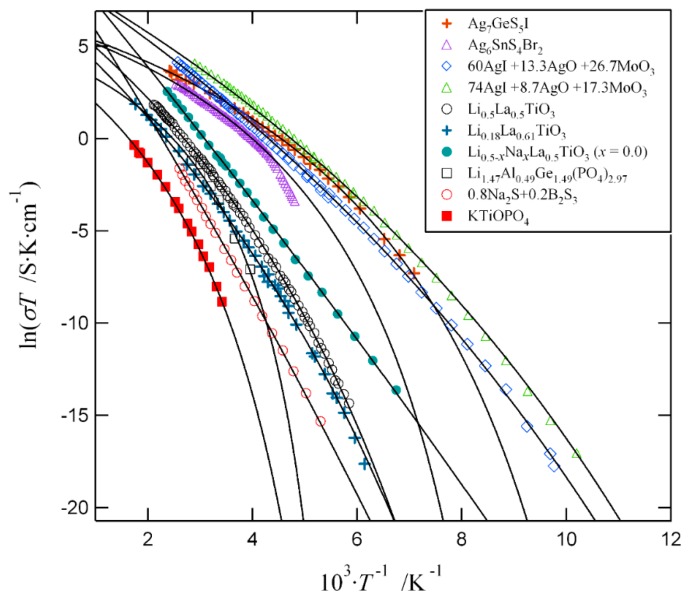
The temperature dependencies of the ionic conductivity in solid electrolytes. The symbols represent the experimental data and the solid curves are described by Equation (5). The values of the model parameters and the sources of the experimental data are given in Table 1.

Equation (5) has been used to analyze the temperature dependence of the ionic conductivity of different materials such as crystalline Ag and Li conductors and some glassy Ag ion conductors [26]. An example of the analysis is shown in Figure 1. From the figure, we can see that the model describes well the experimental data over a wide temperature range of different materials that exhibit Arrhenius- or non-Arrhenius-type behavior. However, it should be mentioned that in some materials, the reproduction of the experimental data is not complete. The main cause of this deviation arises from the neglect of the temperature dependence of $\Delta \tilde{E}\Delta \tilde{Z}$. By taking into account this extension, the temperature dependence of the ionic conductivity can be fitted quite well as shown in the previous report [26].

Analytically, the Arrhenius-type behavior arises when the following condition,

(7) $$T\gg \frac{\Delta \tilde{E}\Delta \tilde{Z}}{R},$$

is satisfied, that is, when the thermal energy is much larger than the bond energy fluctuations. In this case, Equation (5) reduces to the following:

(8) $$\sigma T\approx A_{\sigma T}\exp\left(-\frac{{\tilde{E}}_{0}{\tilde{Z}}_{0}}{RT}\right).$$

In the previous study [26], the obtained values of the model’s parameters were analyzed in connection with the nature of the chemical bond of the materials. There, it was shown that for the case of Ag ion-conducting materials in particular, the magnitude of $\Delta \tilde{E}\Delta \tilde{Z}/{\tilde{E}}_{0}{\tilde{Z}}_{0}$ increased as the compound approached the ionic-covalent borderline defined using concepts of crystal chemistry.

It is interesting to note at this point that the chemical trend of $\Delta \tilde{E}\Delta \tilde{Z}/{\tilde{E}}_{0}{\tilde{Z}}_{0}$ can be separated into two groups [26]. The first group is formed mainly by Ag ion-conducting materials which exhibit the trend mentioned above. The second group is formed mainly by Li ion conductors whose $\Delta \tilde{E}\Delta \tilde{Z}/{\tilde{E}}_{0}{\tilde{Z}}_{0}$ increases as the compound separates from the ionic-covalent borderline. Specifically, the chemical trend of the non-Arrhenius behavior which is reflected in $\Delta \tilde{E}\Delta \tilde{Z}/{\tilde{E}}_{0}{\tilde{Z}}_{0}$ is different in these two groups of materials. The origin of this difference was not clear. Here, a possible interpretation is proposed.

In the formulation of the ionic conductivity described in our model, the temperature dependence of the carrier concentration is not taken into account explicitly. As described above, the temperature effect is absorbed in the values of $\tilde{E}$ and $\tilde{Z}$, or more explicitly, in the values of ${\tilde{E}}_{0}$, ${\tilde{Z}}_{0}$, $\Delta \tilde{E}$, and $\Delta \tilde{Z}$. To reduce the number of free parameters as much as possible, we assume in our analysis that these quantities do not depend on temperature. Physically, this is a crude approximation. If the carrier concentration changes strongly with temperature, the constancy of $\tilde{E}$ and $\tilde{Z}$ does not hold. Interestingly, previous analysis revealed that various Li ion-conducting materials that belong to the second group exhibit a temperature dependent $\Delta \tilde{E}\Delta \tilde{Z}$. Among these, Li_2_TiO_3_ exhibited the strongest temperature dependence [26]. Specifically, there is a possibility that the emergence of two groups in the chemical trends of $\Delta \tilde{E}\Delta \tilde{Z}/{\tilde{E}}_{0}{\tilde{Z}}_{0}$ is related to the temperature dependence of carrier concentration. The clarification of this point is left for a further study.

### 2.2. Ionic Conductivity in Liquids

**Figure 2:**
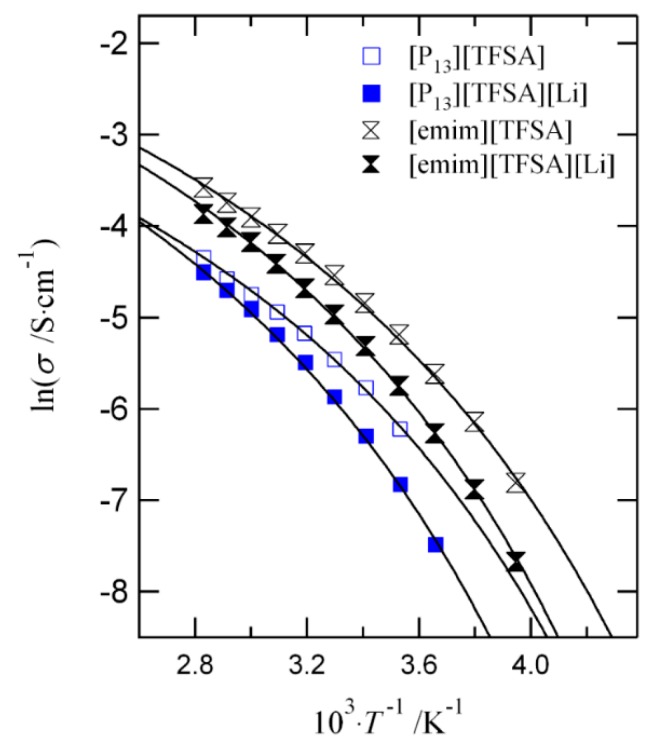
Temperature dependence of the ionic conductivity in ionic liquids [40].

Above, we focused on the description of the non-Arrhenius temperature dependence of the ionic conductivity in solids. A similar expression to that shown in Equation (5) was also used to analyze the conductivity and diffusivity behavior of liquid systems [39,40]. However, for this case, the Stokes–Einstein relation that connects conductivity to viscosity was used. Figure 2 shows an example of the analysis performed for the case of ionic liquids. From the figure, we note that the conductivity in ionic liquids is non-Arrhenius. This type of behavior is not surprising in the case of liquid systems. The important point that we want to stress here is that we now know which factor controls the origin of the non-Arrhenius-like behavior. According to our model [26], it is the magnitude of the fluctuation ΔEΔZ that controls the deviation from the Arrhenius-like behavior. (Note: The symbol tilde is omitted to describe the binding energy E, and the coordination number Z of the structural units in the liquid.)

**Figure 3:**
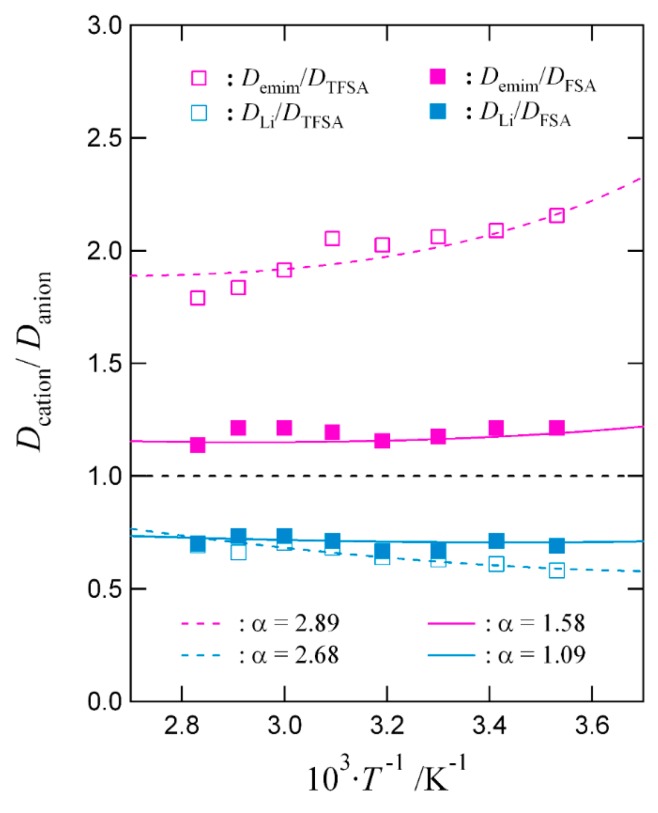
Temperature dependence of the ratio $D_{\mathrm{cation}}/D_{\mathrm{anion}}$ for emim-TFSA, emim-FSA, Li-TFSA, and Li-FSA.

As a related topic, it is interesting to mention that our BSCNF model has been used to relate the degree of dissociation and molecular cooperativity in ionic liquids [41]. Needless to say, the degree of dissociation is an important factor in the application of ionic liquids as electrolytes [42]. An example showing the interplay between the dissociation and molecular cooperativity is shown in Figure 3. The deviation of the ratio between the diffusion constants $D_{\mathrm{cation}}/D_{\mathrm{anion}}$ or $D_{+}/D_{-}$ from unity gives a measure of the dissociation, and it is expressed as follows [41]:

(9) $$\frac{D_{+}}{D_{-}}=\alpha {\left(\frac{1}{N_{B}}\right)}^{\zeta_{+}-\zeta_{-}},$$

where α is a constant, $N_{B}$ is the cooperativity which gives the number of structural units or molecular units that move cooperatively, and $\zeta_{+}$ and $\zeta_{-}$ are quantities that are written in terms of the activation energy of diffusion $E_{D}$ and cooperativity $E_{N_{B}}$ as follows:

(10) $$\zeta_{+}=\frac{E_{D_{+}}}{E_{N_{B}}},\;\mathrm{and}\;\zeta_{-}=\frac{E_{D_{-}}}{E_{N_{B}}}.$$

**Figure 4:**
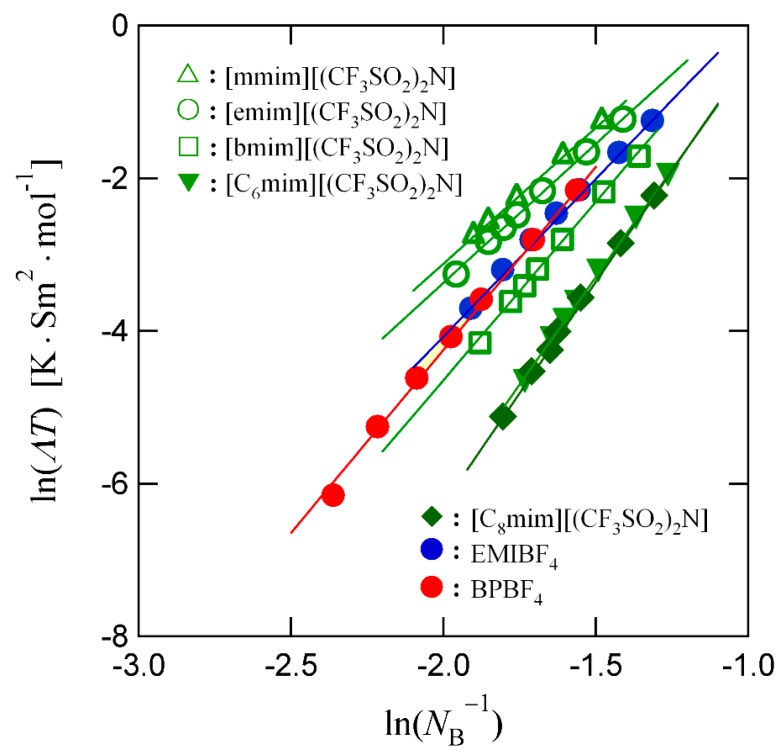
Relation between ln(ΛT) and $\ln\left(N_{B}^{-1}\right)$ in several ionic liquids. Here, Λ is the molar conductivity and $N_{B}$ is the cooperativity.

According to Equation (9), the diffusivity increases with the decease of the cooperativity. This effect is seen more clearly in Figure 4, where the relation between the molar conductivity Λ, which is proportional to the diffusivity, and the cooperativity in several ionic liquids is shown. The physical picture presented here is consistent with the reported experimental results [43].

The above discussion indicates that the cooperativity $N_{B}$ is related intimately to the transport properties such as diffusion and ionic conduction. Analytical expression for the cooperativity has been also obtained in terms of the parameter of the BSCNF model [39]. Therefore, we can say that our model provides a good reference for understanding the complex atomic and molecular dynamics occurring in real systems.

## 3. Possible Size Effect

In this last section, the size effect in the non-Arrhenius mass transport is discussed based on the BSCNF model. In a previous study [29], we have shown that the parameters of our BSCNF model can be related to the parameters of the VFT equation which is used widely in the literature. For instance, the VFT expression of the diffusivity is given by the following:

(11) $$D=D_{0}\exp\left(-\frac{B_{VFT}}{T-T_{0}}\right).$$

The parameters $B_{VFT}$ and $T_{0}$ are related to the parameters of the BSCNF model as follows [29]:

(12) $$B_{VFT}\approx \frac{E_{0}Z_{0}}{R},$$

(13) $$T_{0}\approx \frac{\left|\Delta E\right|\left|\Delta Z\right|}{R}.$$

Based on these relations, we can predict the behavior of the size effect in the non-Arrhenius-type mass transport. Some studies on relaxation behavior in glass forming systems have revealed that $B_{VFT}$ increases and $T_{0}$ decreases with the decrease in the system size. Table 2 shows the example for the cases of a-polystyrene [44] and polyimide copolymer PA66/6I [45]. On the other hand, the opposite trend has been also reported. In Table 2, an example for the case of a poly (methyl ethyl ether)/polystyrene (PVME/PS) blend is shown [46]. According to Equations (12) and (13), the trend exhibited by a-polystyrene and polyimide copolymer PA66/6I suggest that the bond strength $E_{0}Z_{0}$ increases and the fluctuation ΔEΔZ decreases by reducing the size. Specifically, our BSCNF suggests that the degree of non-Arrhenius behavior is suppressed as the size of the system is diminished. For the case of the PVME/PS blend, the opposite trend is expected to occur. This is a simple prediction from the model, stated here for the first time. Although simple, as far as the authors are aware, no previous study has been done regarding this subject. For the experimentalist, it would be interesting to verify if the size effect in the non-Arrhenius behavior really occurs in real systems as predicted by the BSCNF model. However, it should be mentioned that the prediction given here is for an isolated system. For instance, the model does not take into account the role of the grain boundaries or interfaces, which will surely originate other effects [47]. In any case, this is an interesting open subject that deserves further study.

## 4. Conclusions

The description of the temperature dependence of ionic conductivity over a wide range of temperatures is the first step for a deeper understanding of the material’s property. In the present report, it was shown that the ionic conductivity in both solids and liquids can be described based on similar mathematical expressions that were derived from the BSCNF model. Together with the previous findings, the results presented in this paper reconfirm that the BSCNF model provides an effective model to analyze the experimental data. Regarding the size effect, a discussion was given using the VFT parameters of the materials. Within the available data, the BSCNF model predicts that the degree of the non-Arrhenius mass transport behavior can increase or decrease when the system size is diminished. For the case analyzed, the PVME/PS blend is expected to exhibit an opposite trend to those of a-polystyrene and polyamide copolymer PA66/6I.

## Figures and Tables

**Table 1:** Values of the model parameters of Equation (5) for the compounds shown in Figure 1.

Materials	${\tilde{E}}_{0}{\tilde{Z}}_{0}/R\;(K)$	$\Delta \tilde{E}\Delta \tilde{Z}/R\;(K)$	$\ln(A_{\sigma T}/S\cdot K\cdot {\mathrm{cm}}^{-1})$	Ref.
Ag_7_GeS_5_I	910	74	6.28	[6]
Ag_6_SnS_4_Br_2_	950	95	6.19	[8]
60AgI+13.3Ag_2_O+26.7MoO_3_	1850	33	9.29	[5]
74AgI+8.7Ag_2_O+17.3MoO_3_	1600	37	9.20	[5]
Li_0.5_La_0.5_TiO_3_	2200	70	7.31	[7]
Li_0.18_La_0.61_TiO_3_	2500	58	6.79	[10]
Li_0.5-*x*_Na*_x_*La_0.5_TiO_3_ (*x* = 0.0)	3400	10	10.81	[11]
Li_1.47_Al_0.49_Ge_1.49_(PO_4_)_2.97_	1120	156	4.60	[13]
0.8Na_2_S+0.2B_2_S_3_	4340	20	11.39	[1]
KTiOPO_4_	1990	139	4.26	[14]

**Table 2:** Film thickness dependence of the VFT parameters in a-polystyrene, polyamide copolymer PA66/6I, and poly (methyl ethyl ether)/polystyrene (PVME/PS) blend. Numerical data is taken from [44,45,46]. For the polyamide copolymer PA66/6I, the values of $B_{VFT}$ and $T_{0}$ were obtained by fitting the data of diffusion coefficient reported in [45].

Materials	d (nm)	$B_{VFT}$ (K)	$T_{0}$ (K)
a-Polystyrene	18	1887	313
	247	1733	324
Polyamide copolymer PA66/6I	40	1730	292
	56	1569	298
	99	1458	295
	114	1280	302
	556	1076	307
PVME/PS blend	9	537	354
	28	1166	344
	50	1153	327
	84	1212	330
	148	1491	304
